# A Revised View on Growth and Remodeling in the Retinal Vasculature

**DOI:** 10.1038/s41598-019-40135-2

**Published:** 2019-03-01

**Authors:** Ruslan Rust, Lisa Grönnert, Berre Dogançay, Martin E. Schwab

**Affiliations:** 1 Institute for Regenerative Medicine, University of Zurich, 8952 Schlieren, Zurich Switzerland; 2 Department of Health Sciences and Technology, ETH Zurich, 8092 Zurich, Switzerland

## Abstract

The mouse retina provides an excellent model for studying angiogenesis. Recent advancements in high-throughput microscopy and image analysis provide great tools to visualize and describe the complexity of the retinal vascular architecture in a detailed and comprehensive way. Most developmental studies have focused on only a few parameters mostly in the inner-most layers that do not describe the entirety of the three-dimensional vascular network. Here, we analyzed the entire three-dimensional retinal vascular architecture and its growth and remodeling starting from the age of postnatal day 3 to 4 months in mice. We show plexus specific characteristics of the vasculature in terms of vascular tissue fraction, branching and length of the blood vessels, and distance and distribution between single capillaries. Such detailed knowledge is of particular interest, as it has become apparent that disease-specific mechanisms and treatments affect the retinal vasculature often in a plexus specific way.

## Introduction

During development, the vasculature forms a highly complex three-dimensional structure of interconnected blood vessels that provide the developing tissue with essential nutrients, growth factors and oxygen. Any developmental malformations or pathological changes of the network may have fatal consequences in particular for those organs with very high energy demand e.g. nervous tissue including the retina^1,2^.

The metabolic supply of the retina depends on two vascular systems that undergo massive changes and reorganization during development^3^. The uttermost part of the retina, including the photoreceptor layer is nourished by the choroidal vasculature, whereas superficial layers of the retina are supplied by the inner retinal vasculature. It emerges from the central retinal artery and gives rise to three parallel, interconnected vascular networks: the primary plexus located in the nerve fiber layer, and the deep and intermediate plexus lining each side of the inner nuclear layer^4^. The sequential development of each plexus depends on different underlying mechanisms and involves dynamic structural changes. Similarly, it has become apparent that disease related changes can occur in a plexus specific manner^5–7^, and thus may influence the response to treatments^8,9^. Therefore, a detailed assessment of innate layer-specific vascular development is important to study and understand pathological processes, but is missing up to now.

The development of high resolution laser microscopy and 3D imaging provides great new tools to study changes in the vascular architecture in small animals such as mice^10,11^. Computational methods have facilitated the transition from observation-based histology to more quantitative measures, but commercial software is often expensive and requires considerable expertise^12^. Alternatives are open-source packages such as NIH’s ImageJ (FIJI) that also provide excellent plug-ins that are tailored for specific applications to trace and analyze features of the vasculature.

Despite the presence of these diverse imaging and analysis tools, there are only few systematic characterizations of the retinal vascularization in development. The studies are difficult to compare since the choice of relevant parameters, the method of analysis or tissue preparation differ considerably. Moreover, most data are acquired either from maximum projections or exclusively from the uppermost layer and may miss layer specific changes.

Here, we take a new look at the vascular development in the retina at six developmental time points and assess a comprehensive set of biologically relevant architectural parameters, using a reporter mouse for CNS vasculature (Claudin5-eGFP transgenic) on a C57BL/6 background^13^. We show plexus specific analyses of the vascular area fraction, branching, length, distance and distribution of single vessels. The data provide insight into the layer specific pattern remodeling process of the retina and may serve as a reference database for genetic and pharmacologic interventions in retinal vascular development and disease models.

## Results

### Anatomical features of the retinal vasculature

The retina originates as an outgrowth of the forebrain and is therefore considered part of the CNS. Development of the mouse retinal vasculature begins after birth. Being the most accessible part of the CNS, dynamic changes of the vasculature can be easily tracked.

**Figure 1:**
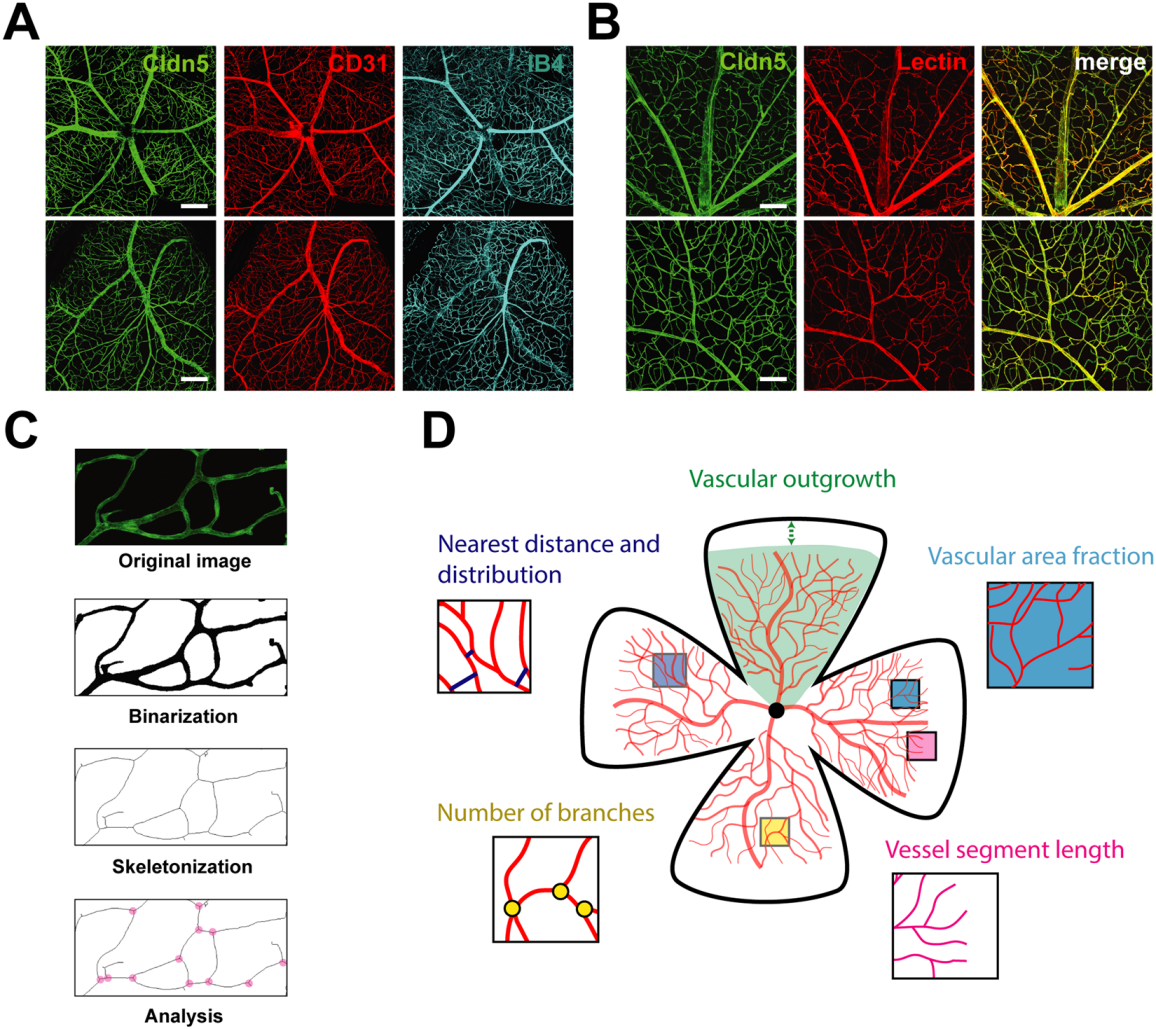
Visualization and image-processing of retinal vasculature. (**A**) Representative images of retinal flatmounts of Cldn5-eGFP reporter mice stained for CD31 and IB4. Scale bar 200 µm. (**B**) Representative images of Cldn5-eGFP^+^ retinal flatmounts after transcardial perfusion with tomato Lectin. Scale bar 100 µm. (**C**) Illustration of the image processing procedure in ImageJ (FIJI). (**D**) Schematic representation of vascular parameters that are assessed in a retinal flatmount. Vascular area fraction: Ratio of area covered by vasculature to total area in the retina.

Initially, we compared the most common procedures for visualization of the developing retinal vasculature. They include the use of immunohistological markers such as antibodies against the vascular adhesion molecule CD31 or Isolectin B4 (Ib4). Alternatives are intravascular perfusion with fluorophore-coupled substances (e.g. tomato Lectin Dylight-594) or the use of genetic mouse models that express a reporter under the control of a vascular endothelium-specific gene (e.g. Cldn5-eGFP mice). Here, we show that all visualization methods result in a uniform vascular staining of the retina (Fig. 1A,B).

To obtain a detailed description of the retinal vasculature, we processed images from Cldn5^+^ retinal flatmounts and established a protocol for a multi-parametric characterization that includes (1) vascular outgrowth (distance from optic nerve head to vascular front), (2) vascular area fraction (ratio of area covered by blood vessels to total retinal area), (3) length of blood vessels (sum of individual vascular segments per mm^2^), (4) branch points (number of arborizations per mm^2^) (5) nearest vessel distance (averaged distance to adjacent vascular segment) and distribution (standard deviation of nearest vessel distance) (Fig. 1C,D).

### Growth and remodeling of the developing vasculature

**Figure 2:**
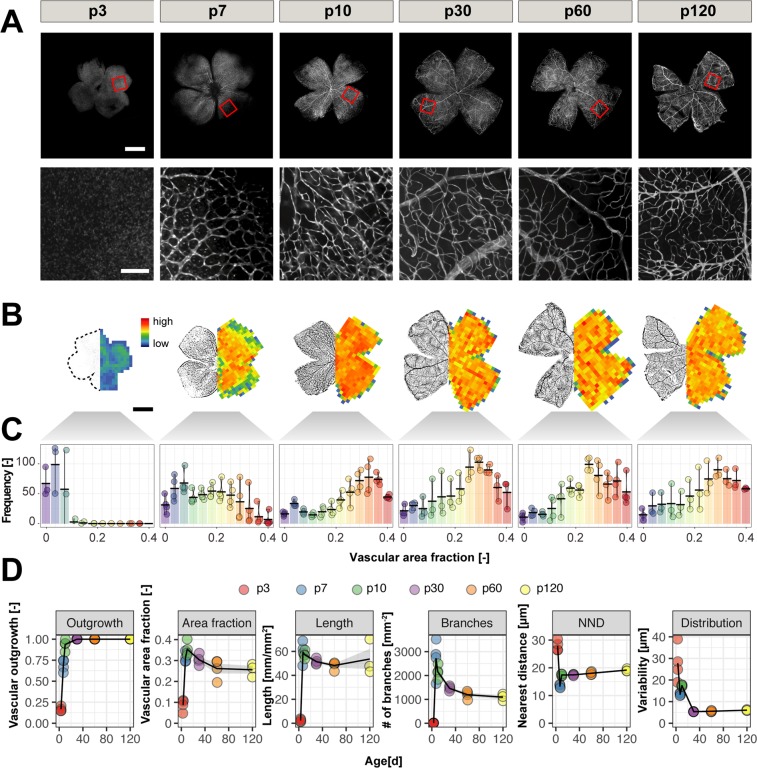
Development of the mouse retinal vasculature from postnatal day p3 to p120. (**A**) Retinal flat mounts of p3, p7, p10, p30, p60 and p120 old mice with close up. Scale bar overview: 1 mm; close up: 100 µm. (**B**) Binarized images of Cldn5-eGFP^+^ retinal flatmounts from p3 to adulthood (p120) (left sides) and corresponding heatmaps of the vascular area fraction (right sides). Scale bar 1 mm. (**C**) Histograms of retinal heatmaps. (**D**) Quantitative evaluation of vascular outgrowth, area fraction, segment length, number of branches, nearest neighbor distance (NND) and variability of NND from p3 to adulthood. The animal number is n = 4 (p3, p7, p10) and n = 3 (p30, p60, p120).

We studied Cldn5^+^ retinal flat mounts at different developmental stages. At postnatal day 3 (p3), the retina was almost completely devoid of blood vessels with only few vessels sprouting from the optic nerve head. At p7 an expanding network becomes visible and reaches the retinal periphery at p10. This is followed by a remodeling process that continues up to 1 month of age, when the retinal vasculature stabilizes (Fig. 2A,B).

Developmental and remodeling processes were described in more detail in maximum projections of whole mount image stacks. The initial outgrowth of the retinal vasculature towards the periphery starts around p3 from the optic nerve head, where it covers a ratio of 0.16 ± 0.01 of the total collapsed retinal area. This is followed by a rapid and continuous expansion of the vasculature that covers the whole retinal area by the age of postnatal day 10. (Fig. 2C). At p10 we observe a peak in the vascular area fraction (0.35 ± 0.17), vessel segment length (57.22 ± 1.53 mm/mm^2^) and branching (2155.24 ± 133.48 branch points/mm^−2^). The distance between the blood vessels is very short (17.41 ± 0.19 µm) but with a rather irregular distribution (15.84 ± 0.54 µm). The initially dense vascular network undergoes substantial remodeling processes until adulthood (p120) characterized by a reduction of the area fraction by 25.7% (p120: 0.26 ± 0.02), segment length by 6.3% (p120: 53.64 ± 8.41) and the number of branch points by 49.3% (p120: 1092.18 ± 82.27). Interestingly, although the distance between the single vessels slightly increases to 10.2% (p120: 19.18 ± 0.27 µm), the distribution of the adult vasculature is 2.6x less variable (p120: 6.04 ± 0.15 µm) compared to the p10 remodeling phase (Fig. 2C).

Altogether, rapid formation of a highly branched vascular network occurs until the age of p10 followed by remodeling processes.

### Development of the primary, intermediate and deep vascular plexus

**Figure 3:**
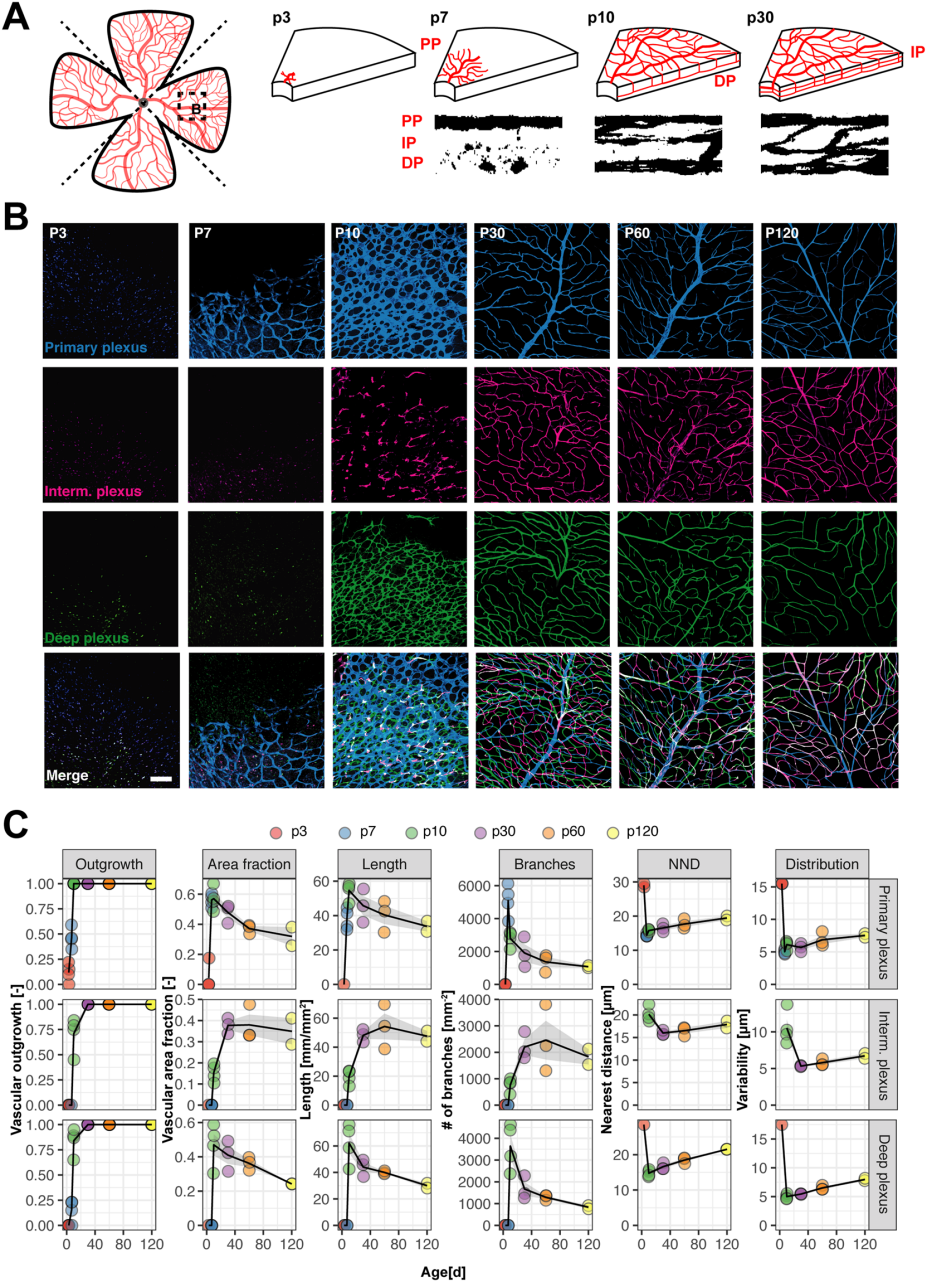
Development of the primary, intermediate and deep vascular plexus. (**A**) Schematic drawings and binarized microscopic images (orthogonal view of retinal wholemount 60x image stack) illustrate the sequential development of the three-layered vascular network in the mouse retina. (**B**) Representative images of the three retinal plexuses at different developmental and adult stages. Scale bar 100 µm. (**C**) Quantitative assessment of vascular parameters reflecting growth and remodeling processes in all three vascular layers. The animal number is n = 4 (p3, p7, p10) and n = 3 (p30, p60, p120).

Analysis of the whole retinal network as described above is useful and provides a reliable and straightforward method to characterize overall structural changes of the radially expanding vascular network. However, it does not provide information on the unique sequential formation and remodeling processes of the three-layered vascular network composed of the primary, intermediate and deep plexus (Fig. 3A).

Image stacks were acquired at mid-radial distance of Cldn5-eGFP^+^ retinal flatmounts with high z-resolution (1 µm). The three separate vascular plexuses, primary, intermediate and deep, were separately projected (‘collapsed’) into one image each, binarized and analyzed as described above. This enabled us to resolve the vascular layers of the retina and detect temporal and structural differences during their formation.

The primary plexus develops first, starting from the optic nerve head and forming a very dense network around the time it reaches the retinal periphery. Its vascular area fraction of 0.57 ± 0.04 at the age of p10 regresses by 41.8% in the adult retina (p120: 0.32 ± 0.06). This remodeling process is also marked by a decline in the blood vessel length (p10: 54.59 ± 2.59 mm/mm^2^; p120: 33.76 ± 2.99 mm/mm^2^), number of branches (p10: 2791.35 ± 229.82 mm^−2^; p120: 1078.21 ± 61.82 mm^−2^) and an increase in the distance between vessels (p10: 15.76 ± 0.17 µm; p120: 19.44 ± 0.58 µm) and its variability (p10: 6.12 ± 0.32 µm; p120: 7.53 ± 0.28 µm) (Fig. 3B,C).

At p10 the deeper and then the intermediate plexus start to grow from the primary plexus penetrating the retina and establishing a laminar network along the outer boundary of the inner nuclear layer. The deep plexus undergoes remodeling similar to the primary plexus, first establishing a dense primitive network that is subsequently reduced (area fraction at p10: 0.47 ± 0.06; p120: 0.24 ± 0.001). The last vascular plexus to develop is the intermediate plexus situated along the inner border of the inner nuclear layer. In contrast to primary and deep plexus, its area fraction increases with time (p10: 0.15 ± 0.02; p120: 0.35 ± 0.06), as do vascular length (p10: 19.66 ± 2.43 mm/mm^2^; p120: 47.58 ± 3.56 mm/mm^2^) and number of branches (p10: 772.80 ± 126.31 mm^−2^; p120: 1828.75 ± 293.05 mm^−2^). The increase in these parameters takes mostly place from p10 to p30 (Fig. 3C). However, some reorganizations in the intermediate plexus might occur between p10 and p30 as indicated by a drop in the high variability of vessel distribution at p10 (10.54 ± 1.14 µm) which drops to half at p30 (5.33 ± 0.05 µm). Although by p30, all three vascular layers were fully formed in the mid-periphery of the retina our analysis shows that some degree of remodeling continues also at later stages. These plexus specific remodeling steps are not detectable in a conventional whole retina analysis.

Since retinal vascularization can differ between strains, we compared single plexus specific data between C57BL/6 and 129S2/Sv mice, another frequently used strain in retinal research, at two time points (p7 and p60). Overall, only minor differences between the vascular development in the two strains were observed. Similarly, at p7 only the primary plexus was detectable in 129S2/Sv mice. Compared to C57BL/6 mice of the same age, the area fraction was reduced by 7.16%, the blood vessel length by 18.4% and the number of branch points by 6.33%. In the adult SV129 retina, at p60, all of the three plexuses were formed. The vascular parameters of both mouse strains contained only minor perceptible differences in a range of max. +/−20%. The most notable alteration is the increase of number of branch points by 25.9% in SV129 animals in the primary plexus (Suppl. Fig. 1, Suppl. Table 4).

Overall, this suggests that although vascular growth and remodeling is known to potentially differ between strains, there are no deviations between C57BL/6 and 129S2/Sv mice to the analysed time points.

### Retinal single plexus analysis provides a more accurate measure of retinal 3D anatomy than whole retinal analysis

**Figure 4:**
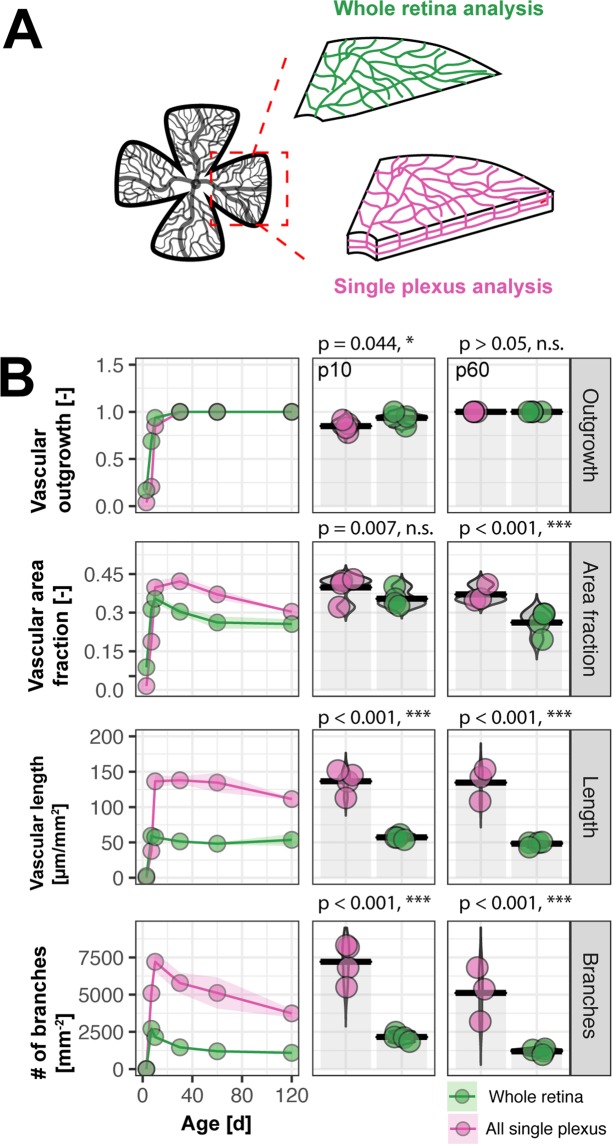
Single plexus analysis reveals more accurate measure of vasculature than whole retina analysis. (**A**) Schematic illustration of whole retina and single plexus analysis. (**B**) Quantification and comparison of vascular outgrowth, area fraction, length and branching between single plexus and whole retina. (C) Tabular overview of vascular parameters with deviations in %. The animal number is n = 4 (p3, p7, p10) and n = 3 (p30, p60, p120).

A major limitation of a collapsed whole retina analysis is the loss of the 3D anatomical properties of the vasculature. Particular changes related to specific processes in a specific layer might not be detectable with this method. We compared the collapsed whole retina tissue analysis with an extrapolation of the three single plexuses of the developing vasculature. To do this, we summed up the length and the branching of the vasculature and averaged the vascular area fraction and the distance and distribution of the vasculature within the single plexus (Fig. 4A). Next, we compared the data to all developmental time points with the whole tissue analysis. In general, we observed a clear underestimation of most vascular parameters in the whole tissue analysis. Especially, the number of branches and the length of the vasculature deviated significantly between the single plexus analysis and the whole retinal analysis. The difference was up to 234.38% (p10), or respectively 328.03% (p60) for the number of branch points and 138.40% (p10), or respectively 99.98% (p60) for the length of the vasculature (Fig. 4B, Tables 1, 2).

**Table 1:** Differences in vascular parameters in developmental retina (p10).

Parameter	Whole retina analysis	Single plexus analysis	Deviation
Outgrowth	0.94	0.85	−9.57
Area fraction	0.35	0.40	+14.29
Length	57.22	136.41	+138.40
Branches	2155.25	7206.81	+234.38
Nearest distance	17.41	16.89	−2.97

**Table 2:** Differences in vascular parameters in the adult retina (p60).

Parameter	Whole retina analysis	Single plexus analysis	Deviation
Outgrowth	1	1	0
Area fraction	0.26	0.37	+42.31
Length	48.23	134.66	+99.98
Branches	1194.79	5114.07	+323.03
Nearest distance	18.04	17.54	−2.77

In summary, it suggests that the single plexus analysis provides new insights into the vascular development with a more accurate measure compared to collapsed whole retina analysis. This approach might be especially relevant when it comes to analysis of pathological changes in the retina due to genetic manipulations or in a therapeutic experimental setting since the molecular basis of the plexus formation considerably differs and may not be properly described in a collapsed whole retinal analysis.

## Discussion

Due to its postnatal development and easy accessibility, the mouse retinal vasculature is an excellent model to study vascular growth and remodeling during development and disease. Advances in imaging techniques and analysis methodology contribute to the understanding of the development of the retinal vasculature and could help elucidate pathological vascular alterations. However, besides comprehensive descriptive analyses most available studies lack comparable quantitative measurements. Here, we propose a quantitative description of the vascular development in the postnatal retina of C57BL/6 mice by taking advantage of open source analysis tools that allow to obtain relevant vascular parameters automatically and efficiently. We describe a gradual growth of the retinal vasculature in the first postnatal days, and dynamic remodeling processes from the age of 10 days to 1–4 months. Furthermore, we show that a plexus specific description of the primary, deep and intermediate plexus reveals more details than a collapsed whole retina analysis. This set of data can serve as a basis study of genetic or pharmacological manipulations of the retinal vasculature.

Since retinal vascularization occurs in a timely highly coordinated fashion and can vary considerably between strains, it must be noted that the presented data only apply for the retinal vascular development in C57BL/6 mice. Although we did not discover major changes between C57BL/6 and SV129 mice in our study, there might be considerable deviations in the time course and vascular anatomy in other strains especially in those with retinal degeneration. In BALB/c mice, for example, retinal vascular development is delayed in comparison to C57BL/6 mice, which are most commonly used in the field of retinal vascular research^14^. So far, however, a detailed quantitative description of the retinal vascular network of C57BL/6 mice was missing. Methodological approaches were not standardized and often done in a non-automated fashion, making analysis work intensive and prone to errors. So far, quantitative analysis of the vascular network of the retina was not standardized and was often done in a non-automated fashion, making analysis work intensive and prone to errors. Although there are a few easily accessible and user-friendly software tools^15^, most studies quantitatively rely on the “vascular density” of collapsed whole retinae, thereby missing important biological parameters^16^. However, in the developing retinal vasculature, the branching and length of blood vessels are more relevant for actual oxygen and nutrient exchange as already postulated by Murray’s law in 1926^17,18^. Furthermore, the distance between blood vessels and its variability may contribute important information on homogenous tissue oxygenation, as they indicate local hypoxic areas that are not entirely represented by the overall mean vascular density.

By implementing these measures in the developing retinal vasculature, we show the growth of the three layers in the inner retina. Our results indicate that the three vascular plexuses are generated in a heterochronous way. The formation of each plexus has its specific characteristics, probably reflecting different underlying molecular mechanisms of growth regulation and guidance of endothelial cell migration^4,19^. Our findings are in accordance with previous studies^3,20^. However, most of these studies, often interested in disease modeling or treatment approaches, focus on the first weeks of life without addressing subsequent remodeling processes and provide only few quantitative measures. By our quantitative assessment, we observed substantial remodeling processes that take place long after the formation of the three plexuses^21,22^.

Apart from the choice of parameters, the three-dimensional structure of the retina is important and not considered in most studies performing collapsed whole retina analysis. Therefore, we compared data from whole retinal analysis to single plexus analysis. Overall, both methods provide valuable tools to describe the vasculature. However, we suggest that studies focusing on absolute values including total number of branches or total length of the vasculature would profit from a single plexus analysis. Moreover, pharmacological interventions might not be described properly by a whole retinal analysis since underlying mechanisms of vascular growth in this plexus are not identical. In development, initial vascularization of the retina is sculpted by different retinal cell types with a dominant role for neurons and astrocytes. Retinal ganglion cells and an astrocytic framework are primarily involved in primary plexus formation^23,24^, whereas the growth of the intermediate and deep plexus critically depends on the location of VEGF releasing neuronal populations residing in the distinct retinal laminae^25^. Accordingly, differences in molecular pathways showed plexus specific alterations that e.g. include the Wnt canonical pathway. Mutations in Norrin, Frizzled, Tetraspanin12 or LRP5 led to primary plexus formation with a lack or abnormalities in intermediate layer and deep plexus layer^5–7^. These findings clearly indicated that molecular mechanisms between the vascular layers differ and so may a pharmaceutical treatment introduce different responses within the single layers.

In conclusion, we provide a simple protocol to rapidly and precisely assess various vascular parameters over time which could be relevant for studies addressing retinal development or disease. Moreover, our study can be used as a reference library containing detailed measures of retinal vasculature within single plexus resolution that could also serve as a dataset for validation of alternative analysis tools or computer models that simulate vascular growth.

## Methods

### Experimental design

The aim of the study was to show various aspects of vascular development in the three-vascular plexus of the retina. We chose different points in time during development and adulthood ranging from postnatal day 3 to adulthood (4 months). A total number of 21 mice was used for the study. We analyzed the time points p3 (n = 4), p7 (n = 4), p10 (n = 4), p30 (n = 3), p60 (n = 3), p120 (n = 3). We analyzed different biologically relevant aspects of vascular development and remodeling. They include vascular area fraction, branching and length of the vasculature, distance and distribution between single vessels. Furthermore, we compared a whole tissue retinal analysis approach to analysis of the single vascular plexuses. All animals are presented in this study; no statistical outliers were excluded. Data was acquired blinded. The analysis of the data was performed by different investigators that were blinded in all steps of analysis.

### Animals

We used wildtype and Claudin-eGFP C57BL/6^13^ mice. Animals were maintained at the Brain Research Institute Zurich on a 12 h light/dark cycle with food and water provided *ad libitum*. Mice were housed in standard Type II/III cages at least in pairs. Animals used were 3 days to 4-month-old males and females. All experiments were conducted in accordance with the applicable national regulations and approved by the Cantonal Veterinary Department of Zurich.

### Tissue collection and processing

Animals were deeply anaesthetized by intraperitoneal injection of pentobarbital (150 mg/kg bodyweight) and eyes enucleated using curved forceps. For retinal flatmount preparation, eyes were prefixed with 4% PFA for 20 min at 4 °C following excision of the cornea, lens, sclera and vitreous and isolation of the retina. Retinas were flatmounted on permeable cell culture inserts (BD Falcon) and sequentially fixed with 4% PFA from both sides for 20 min at 4 °C, respectively. For immunohistochemical staining retinal flatmounts were washed with 0.1 M phosphate buffer (PB) and then incubated with a blocking and permeabilization solution for 30 min at RT shaking. Tissue was incubated with primary antibodies against CD31 (rat, 1:100, BD Biosciences) and Isolectin B4 (1:100, Vector Labs), overnight at 4 °C. The next day the tissue was washed and incubated with corresponding secondary antibodies conjugated to Cy3 (Thermo Fischer) for 2 h at RT. All antibodies were diluted in blocking and permeabilization solution (TNB, 0.1% TBST, 3% normal goat serum). Flatmounts were mounted in 0.1 M PB on Superfrost Plus^TM^ microscope slides (Thermo Fisher) and coverslipped using Mowiol.

### Vascular tracing with tomato lectin

Animals were deeply anaesthetized by intraperitoneal injection of pentobarbital (150 mg/kg bodyweight) and subsequently received an intracardial injection of 50 µl of 1 mg/ml tomato lectin conjugated to DyLight594. After 2 minutes, eyes were removed and processed as described above.

### Fluorescence microscopy

Wholemount retinal images were acquired using an Axio Scan.Z1 slide scanner (Zeiss) equipped with a 10x objective. For single plexus visualization 20x image stacks were obtained at mid-radial distance of every quadrant of Cldn5-eGFP^+^ retinal flatmounts using an Olympus FV1000 laser scanning confocal microscope with a Z-resolution of 1 µm. Images were processed using Zen 2 software, Fiji (ImageJ) and Adobe Illustrator CC.

### Image Analysis

Images were analyzed with ImageJ (FIJI). Images were converted into 8-bit format and thresholded with the AdaptiveThreshold plugin to get a binarized image. A median of 0.5 pixels was applied to remove noise. In wholemount images the region of interest (ROI) was manually selected. Single plexus analysis was performed in four images per animal and plexuses were separately projected in one image, binarized and analyzed for all parameters; (1) Area fraction: The ratio of pixels in ROI that have been selected (are not zero) to total number of pixels in ROI. (2) Vascular length: The image was skeletonized and analyzed with the plugin Skeleton length tool. The length of all structures in the ROI was summed up (3) Number of branches was assessed by the Analyze Skeleton tool. (4) Distance and variability of the vessels was calculated by NND tool that calculated the minimal distance between the single vessels. From this we calculated the mean and the standard deviation to get information about the average distance and variability in distribution between the vessels in ROI. The values vascular length, number of branches were normalized to the total area of region of interest. Heat maps of whole tissues were performed by calculating the area fraction in single ROIs across the whole image and adjusting a custom-made LUT to the area fraction values for visual representation. The complete FIJI script is available on request. All raw data is attached to the manuscript (Supplementary Tables 1–3).

### Statistical analysis

All data is presented as means ± standard error of the mean (s.e.m.). All data was tested for normal distribution by using the Shapiro-Wilk test. Anatomical changes over time were assessed using one way ANOVA measures followed by Dunnett’s post hoc test. Whenever two groups were compared; (1) Normally distributed data was tested for differences with a two-tailed unpaired one-sample t-test; (2) Non-normally distributed data was tested with a Mann-Whitney U test. Statistical analysis was performed using RStudio. The level of significance was set at *P < 0.05, **P < 0.01, ***P < 0.001.

## Supplementary information

Supplementary Material
