# Supplementary material for: A Revised View on Growth and Remodeling in the Retinal Vasculature
Source: Sci Rep. 2019 Mar 1;9:3263. doi: 10.1038/s41598-019-40135-2 (PMC6397250; doi:10.1038/s41598-019-40135-2)
Supplement: Supplementary file 1 — Supplementary Material [file 41598_2019_40135_MOESM1_ESM.pdf]

SUPPLEMENTARY MATERIAL TO ARTICLE:

TITLE

A Revised View on Growth and Remodeling in the Retinal Vasculature

AUTHORS

Ruslan Rust[1,2,*], Lisa Grönnert[1], Berre Dogançay [2], Martin E. Schwab[1,2]

AFFILIATIONS

[1] Institute for Regenerative Medicine, University of Zurich, 8952 Schlieren, Zurich, Switzerland,

[2] Dept. of Health Sciences and Technology, ETH Zurich, 8092 Zurich, Switzerland

* Corresponding author

CORRESPONDANCE

Ruslan Rust
Laboratory of Neural Regeneration and Repair
Institute for Regenerative Medicine (IREM)
University of Zurich, Campus Schlieren
Wagistrasse 12
8952 Schlieren / Zurich, Switzerland
rust@irem.uzh.ch, +41 44 63 53215

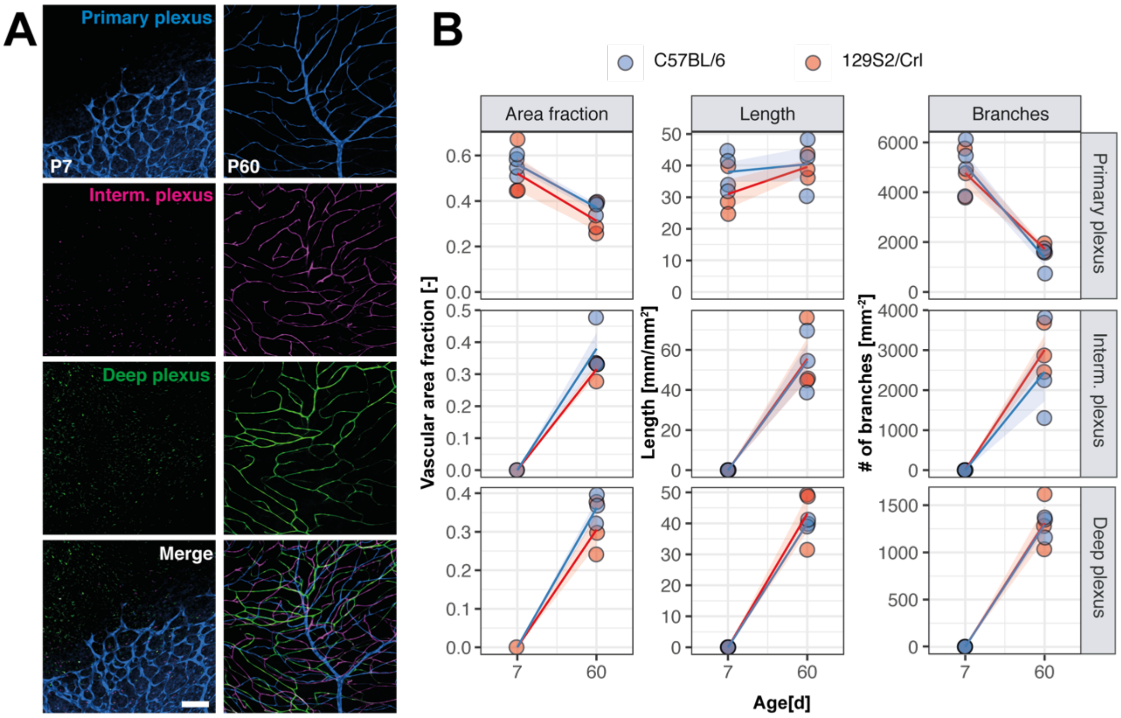

Supplementary Figure 1: Vascular development in 129S2/Sv mice is similar to C57BL/6J. (A) Representative images of three retinal plexuses of 129S2/Sv mouse retinas at p7 and p60. Scale bar 100 µm. (B) Quantitative assessment of vascular parameters reflecting growth and remodeling processes in all three vascular layers. The animal number is for C57BL/6 (p7; n=4, p60; n=3) and for 129S2/Sv (p7, p60; n = 3).

Supplementary Table 1: Vascular parameter for whole retinal analysis.

age of animals	vascular parameter	mean	sd	sem
p3	Area fraction	0.087	0.028	0.014
p7	Area fraction	0.314	0.023	0.012
p10	Area fraction	0.354	0.034	0.017
p30	Area fraction	0.304	0.025	0.012
p60	Area fraction	0.262	0.047	0.024
p120	Area fraction	0.256	0.032	0.018
p3	Branch points	8.362	5.365	2.682
p7	Branch points	2705.083	773.207	386.604
p10	Branch points	2155.241	266.955	133.478
p30	Branch points	1460.173	94.288	47.144
p60	Branch points	1194.795	160.336	80.168
p120	Branch points	1092.183	142.495	82.269
p3	Distribution	27.798	8.397	4.199
p7	Distribution	13.395	0.570	0.285
p10	Distribution	15.841	0.312	0.544
p30	Distribution	5.344	0.089	0.044
p60	Distribution	5.538	0.315	0.157
p120	Distribution	6.041	0.256	0.148
p3	Length	2.314	1.180	0.590
p7	Length	59.392	8.655	4.327
p10	Length	57.225	3.071	1.535
p30	Length	51.411	2.303	1.152
p60	Length	48.231	3.275	1.637
p120	Length	53.646	14.568	8.411
p3	NND	27.941	1.880	0.940
p7	NND	13.395	0.570	0.285
p10	NND	17.411	0.376	0.188
p30	NND	17.450	0.254	0.127
p60	NND	18.035	0.554	0.277
p120	NND	19.178	0.461	0.266
p3	Outgrowth	0.168	0.028	0.014
p7	Outgrowth	0.689	0.069	0.035
p10	Outgrowth	0.936	0.064	0.032
p30	Outgrowth	1.000	0.000	0.000
p60	Outgrowth	1.000	0.000	0.000
p120	Outgrowth	1.000	0.000	0.000

Supplementary Table 2: Vascular parameter for single plexus analysis.

age of animals	vascular parameter	plexus	mean	sd	sem
p3	Area fraction	PP	0.044	0.088	0.044
p7	Area fraction	PP	0.561	0.042	0.021
p10	Area fraction	PP	0.570	0.077	0.038
p30	Area fraction	PP	0.478	0.062	0.036
p60	Area fraction	PP	0.371	0.030	0.017
p120	Area fraction	PP	0.319	0.088	0.062
p3	Branch points	PP	0.000	0.000	0.000
p7	Branch points	PP	5092.434	967.008	483.504
p10	Branch points	PP	2791.349	459.630	229.815
p30	Branch points	PP	1911.594	913.258	527.270
p60	Branch points	PP	1365.896	547.269	315.966
p120	Branch points	PP	1078.209	87.432	61.824
p3	Distribution	PP	-	-	-
p7	Distribution	PP	5.034	0.375	0.188
p10	Distribution	PP	6.118	0.640	0.320
p30	Distribution	PP	5.682	0.638	0.368
p60	Distribution	PP	6.863	1.118	0.646
p120	Distribution	PP	7.531	0.398	0.281
p3	Length	PP	0.000	0.000	0.000
p7	Length	PP	37.928	6.057	3.029
p10	Length	PP	54.592	5.189	2.594
p30	Length	PP	45.757	9.680	5.589
p60	Length	PP	40.376	9.184	5.302
p120	Length	PP	33.761	4.233	2.993
p3	NND	PP	-	-	-
p7	NND	PP	14.395	0.448	0.224
p10	NND	PP	15.756	0.333	0.167
p30	NND	PP	16.528	1.184	0.683
p60	NND	PP	17.667	1.418	0.819
p120	NND	PP	19.439	0.822	0.581
p3	Outgrowth	PP	0.118	0.093	0.046
p7	Outgrowth	PP	0.463	0.098	0.049
p10	Outgrowth	PP	1.000	0.000	0.000
p30	Outgrowth	PP	1.000	0.000	0.000
p60	Outgrowth	PP	1.000	0.000	0.000
p120	Outgrowth	PP	1.000	0.000	0.000
p3	Area fraction	IP	0.000	0.000	0.000
p7	Area fraction	IP	0.000	0.000	0.000
p10	Area fraction	IP	0.154	0.040	0.020
p30	Area fraction	IP	0.377	0.037	0.021
p60	Area fraction	IP	0.380	0.084	0.049
p120	Area fraction	IP	0.349	0.087	0.062
p3	Branch points	IP	0.000	0.000	0.000
p7	Branch points	IP	0.000	0.000	0.000
p10	Branch points	IP	772.799	252.617	126.309
p30	Branch points	IP	2199.282	517.648	298.864
p60	Branch points	IP	2456.469	1268.876	732.586
p120	Branch points	IP	1828.751	414.429	293.045
p3	Distribution	IP	-	-	-
p7	Distribution	IP	-	-	-

p10	Distribution	IP	10.539	2.273	1.136
p30	Distribution	IP	5.328	0.080	0.046
p60	Distribution	IP	5.794	0.466	0.269
p120	Distribution	IP	6.721	0.471	0.333
p3	Length	IP	0.000	0.000	0.000
p7	Length	IP	0.000	0.000	0.000
p10	Length	IP	19.656	4.863	2.432
p30	Length	IP	48.138	4.357	2.516
p60	Length	IP	54.269	15.392	8.887
p120	Length	IP	47.578	5.032	3.558
p3	NND	IP	-	-	-
p7	NND	IP	-	-	-
p10	NND	IP	20.161	1.601	0.801
p30	NND	IP	16.001	0.528	0.305
p60	NND	IP	16.497	1.019	0.589
p120	NND	IP	17.887	1.092	0.772
p3	Outgrowth	IP	0.000	0.000	0.000
p7	Outgrowth	IP	0.000	0.000	0.000
p10	Outgrowth	IP	0.708	0.176	0.088
p30	Outgrowth	IP	1.000	0.000	0.000
p60	Outgrowth	IP	1.000	0.000	0.000
p120	Outgrowth	IP	1.000	0.000	0.000
p3	Area fraction	DP	0.000	0.000	0.000
p7	Area fraction	DP	0.000	0.000	0.000
p10	Area fraction	DP	0.470	0.122	0.061
p30	Area fraction	DP	0.410	0.089	0.051
p60	Area fraction	DP	0.362	0.038	0.022
p120	Area fraction	DP	0.242	0.001	0.001
p3	Branch points	DP	0.000	0.000	0.000
p7	Branch points	DP	0.000	0.000	0.000
p10	Branch points	DP	3642.664	1055.190	527.595
p30	Branch points	DP	1670.894	541.653	312.723
p60	Branch points	DP	1291.707	116.893	67.488
p120	Branch points	DP	835.859	104.919	74.189
p3	Distribution	DP	-	-	-
p7	Distribution	DP	-	-	-
p10	Distribution	DP	5.001	0.421	0.211
p30	Distribution	DP	5.442	0.064	0.037
p60	Distribution	DP	6.503	0.391	0.226
p120	Distribution	DP	7.976	0.287	0.203
p3	Length	DP	0.000	0.000	0.000
p7	Length	DP	0.000	0.000	0.000
p10	Length	DP	62.165	14.984	7.492
p30	Length	DP	44.039	6.404	3.697
p60	Length	DP	40.002	1.103	0.637
p120	Length	DP	30.019	2.554	1.806
p3	NND	DP	-	-	-
p7	NND	DP	-	-	-
p10	NND	DP	14.743	0.931	0.466
p30	NND	DP	16.591	0.961	0.555
p60	NND	DP	18.464	0.863	0.498
p120	NND	DP	21.523	0.006	0.004
p3	Outgrowth	DP	0.000	0.000	0.000

p7	Outgrowth	DP	0.153	0.108	0.054
p10	Outgrowth	DP	0.840	0.131	0.066
p30	Outgrowth	DP	1.000	0.000	0.000
p60	Outgrowth	DP	1.000	0.000	0.000
p120	Outgrowth	DP	1.000	0.000	0.000

Supplementary Table 3: Extrapolation of the three single plexuses of the developing vasculature.

age of animals	vascular parameter	mean	sd	sem
p3	Area fraction	0.014	0.029	0.014
p7	Area fraction	0.187	0.014	0.007
p10	Area fraction	0.397	0.051	0.025
p30	Area fraction	0.421	0.029	0.017
p60	Area fraction	0.370	0.033	0.019
p120	Area fraction	0.303	0.000	0.000
p3	Branch points	0.000	0.000	0.000
p7	Branch points	5092.434	967.008	483.504
p10	Branch points	7206.812	1318.135	659.067
p30	Branch points	5781.770	1198.302	691.840
p60	Branch points	5114.072	1808.086	1043.899
p120	Branch points	3742.819	396.942	280.680
p3	Distribution	15.175	2.196	1.098
p7	Distribution	5.034	0.375	0.188
p10	Distribution	7.219	0.827	0.414
p30	Distribution	5.484	0.227	0.131
p60	Distribution	6.387	0.463	0.267
p120	Distribution	7.409	0.194	0.137
p3	Length	0.000	0.000	0.000
p7	Length	37.928	6.057	3.029
p10	Length	136.412	16.787	8.394
p30	Length	137.933	9.181	5.301
p60	Length	134.647	23.661	13.661
p120	Length	111.357	1.755	1.241
p3	NND	29.875	2.136	1.068
p7	NND	14.395	0.448	0.224
p10	NND	16.887	0.403	0.201
p30	NND	16.373	0.601	0.347
p60	NND	17.543	0.864	0.499
p120	NND	19.616	0.636	0.450
p3	Outgrowth	0.039	0.031	0.015
p7	Outgrowth	0.205	0.051	0.026
p10	Outgrowth	0.849	0.057	0.029
p30	Outgrowth	1.000	0.000	0.000
p60	Outgrowth	1.000	0.000	0.000
p120	Outgrowth	1.000	0.000	0.000

Supplementary Table 4: Vascular parameter for single plexus analysis in 129S2/Ctrl mice.

Groups	Parameter	Plexus	Strain	mean	sd	sem
p7	Area fraction	DP	129SV	0.000	0.000	0.000
p60	Area fraction	DP	129SV	0.305	0.068	0.039
p7	Area fraction	IP	129SV	0.000	0.000	0.000
p60	Area fraction	IP	129SV	0.316	0.033	0.019
p7	Area fraction	PP	129SV	0.521	0.130	0.075
p60	Area fraction	PP	129SV	0.313	0.074	0.043
p7	Branchpoints	DP	129SV	0.000	0.000	0.000
p60	Branchpoints	DP	129SV	1310.411	293.194	169.275
p7	Branchpoints	IP	129SV	0.000	0.000	0.000
p60	Branchpoints	IP	129SV	3003.285	626.486	361.702
p7	Branchpoints	PP	129SV	4769.920	983.515	567.833
p60	Branchpoints	PP	129SV	1719.688	203.924	117.736
p7	Length	DP	129SV	0.000	0.000	0.000
p60	Length	DP	129SV	43.138	10.106	5.835
p7	Length	IP	129SV	0.000	0.000	0.000
p60	Length	IP	129SV	55.548	17.829	10.293
p7	Length	PP	129SV	30.950	7.845	4.529
p60	Length	PP	129SV	39.504	3.821	2.206
